# Cryopreservation of ferret (*Mustela putorius furo*) sperm collected by rectal massage and electroejaculation: Comparison of a decelerating and an accelerating freezing rate protocol

**DOI:** 10.1002/vms3.362

**Published:** 2020-10-11

**Authors:** Adolfo Toledano‐Díaz, Cristina Castaño, Rosario Velázquez, Paula Bóveda, Antonio López‐Sebastián, Eva Martínez‐Nevado, Silvia Villaverde‐Morcillo, Milagros C. Esteso, Julián Santiago‐Moreno

**Affiliations:** ^1^ Dpto. Reproducción Animal INIA Madrid Spain; ^2^ Zoo‐Aquarium Madrid Spain

**Keywords:** cooling rate, electroejaculation, mustelids, sperm

## Abstract

The domestic ferret (*Mustela putorius furo*) provides a good model for developing new reproductive technologies for use with threatened related species. Such technologies could also be used in the reproductive management of this pet species. The present work reports an improved freezing protocol for ferret sperm. Semen was collected by electroejaculation plus rectal massage (in an attempt to reduce the electrical stimulation necessary) from five adult male ferrets, and then subjected to one of two freezing protocols: (a) from 5 to −35°C at 40°C/min, then from −35 to −65°C at 17°C/min, and finally from −65 to −85°C at 3°C/min—a decelerating freezing rate; and (b) from 5 to − 10°C at 5°C/min, and then from −10 to −130°C at 60°C/min—an accelerating freezing rate. After thawing, the viability and acrosomal integrity of the sperm frozen via the two‐step accelerating method were better than those frozen via the three‐step decelerating method (43.3 ± 3.5% and 71.2 ± 3.4% compared with 29.7 ± 3.7% and 58.8 ± 3.4% respectively; *p* < .05). No differences were seen between the methods with respect to sperm motility variables; most sperm (>90%) remained static with both freezing methods. In conclusion, although the method with accelerating freezing rate was associated with better post‐thaw sperm viability and acrosome integrity values, neither of the two freezing methods tested provided adequate motility results after thawing. Combining rectal massage with electrical stimuli seemed to reduce the number of the latter required for successful sperm collection.

## Graphical abstract

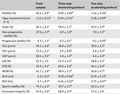

## 1 INTRODUCTION

The genus *Mustela* is represented by 19 species worldwide, but only the European mink (*Mustela lutreola*) is listed as Critically Endangered (IUCN Red List, 2020). Historically, this species ranged from Finland, eastward to beyond the Ural Mountains, and southward to northern Spain and the Caucasus, but over the last 150 years it has experienced severe population decline (Maran, 1999, 2007). Several conservation associations and the IUCN have recommend ex situ conservation programs begin, including captive breeding programs that involve the use of reproductive technologies as required (Holt & Pickard, 1999). However, techniques suited for use with *Mustela lutreola* are needed.

Reproductive techniques for use with a target species can be developed using phylogenetically related model species. For example, black‐footed ferret (*Mustela nigripes*) conservation programs in the USA have made use of non‐endangered domestic ferrets (Pukazhenthi & Wildt, 2004). The taxonomic proximity of ferrets to the European mink (Amstislavsky et al., 2008) means they may also serve as a model for the latter. Any assisted reproduction techniques developed might, of course, also be of use in the reproductive management of ferrets themselves as pets.

Domestic ferrets (*Mustela putorius furo*) are seasonal breeders in which reproductive activity is stimulated by a long‐day photoperiod (Bissonette, 1932, 1935; Hammond & Marshall, 1930). Testicular activity, which begins in December or January, gradually increases and peaks from April to June (Neal et al., 1977). The males enter sexual quiescence from August to December (Ishida, 1968; Miller et al., 1988). Semen must therefore be collected during the period of reproductive activity (van der Horst et al., 2009).

Most electroejaculation protocols used with mustelids are based on that developed by Wildt et al. (1989), and require anaesthesia and several sets of electrical stimuli. An electroejaculation protocol that reduces the number and intensity of electrical stimuli will decrease discomfort and pain caused to the male, thereby improving animal welfare. Transrectal massage close to the sexual accessory glands might induce adrenergic stimuli that would favour the ejaculation. Moreover, massage directed specifically at the ampulla of the vas deferens may hasten sperm emission by inducing endogenous oxytocin release (Palmer el at., 2004). The effectiveness of transrectal massage of accessory sexual glands to recover sperm has been demonstrated in humans (Fahmy et al., 1999), non‐human primates (Gadea et al., 2019) and wild ruminants (Santiago Moreno et al., 2013).

Several studies on ferret sperm production and quality, in which conventional sperm variables were assessed, have been reported (Howard et al., 1991, 2016; Santymire et al., 2006; Wildt et al., 1989), but less work has been done in the area of kinematic sperm variables (van der Horst et al., 2009).

Semen cryopreservation methods have been developed for the ferret, but these have met with varying levels of success. As with many semen freezing protocols for carnivorous species, TEST diluent (Irvine Scientific) is used in mustelid sperm processing as it seems to provide better protection (van der Horst et al., 2009). The freezing rates employed depend on the exact technique used, which may involve the use of dry ice blocks, bio‐freezing, or nitrogen vapour immersion, etc. Some authors have reported good results after dropping sperm onto dry ice to produce pellets (Howard et al., 2016; Leigh, 2010; Santymire et al., 2007). Frozen‐thawed ferret semen has returned up to 70% pregnancy rates (Howard et al., 1991). The cooling rate is one of the key factors for sperm freezing success. As extracellular ice formation takes place, the cells and the dissolved salts are excluded from the ice and become concentrated between the growing ice masses. The osmotic strength of this unfrozen fraction produces the sperm shrinkage. Too low cooling rate may cause excessive dehydration of the cell and subsequent membrane damage (Katkov, 2012). Furthermore, the velocity at which ice forms and the distribution and size of ice crystals during sperm cryopreservation are strongly dependent on the cooling rate (Bóveda et al., 2020).

The aim of the present work was to improve ferret semen cryopreservation by examining the effect of two freezing protocols, one with a decelerating and one with an accelerating freezing rate, on the quality of frozen‐thawed sperm. It is hypothesized that the use of acceleration cooling rates, using slow initial speeds to avoid cold shock and severe cellular dehydration, followed by faster cooling rates can produce less damage to sperm cells and better post‐thaw quality, as has been demonstrated in the freezing of sperm from other species (Esteso et al., 2018; Galarza et al., 2019). Transrectal massage was combined with electrical stimuli in a preliminary attempt to reduce the need for the latter.

## 2 MATERIALS AND METHODS

### 2.1 Animals

Sperm was collected from five male ferrets (27 months old; body weight 900–1,200 g) housed individually in cages (90 × 90 × 42 cm) at the INIA Department of Animal Reproduction (Madrid, 40° 25'N). All had ad libitum access to food (Hurones Arion Ferret, Piensos Alonso, Navalcarnero, Madrid) and water. Artificial light was provided by fluorescent bulbs. The light cycle was regulated to provide 15 hr of light and 6 hr of darkness (long‐day photoperiod).

All animals were managed in a manner consistent with the Spanish Policy for Animal Protection (RD 53/2013), which conforms to European Union Directive 2010/63/EU regarding the protection of animals used in scientific experiments.

### 2.2 Semen collection and assessment of fresh semen variables

Following a 24‐hr fast without water, the ferrets were manually restrained and anaesthetized with 5 mg/kg intramuscular (i.m.) ketamine hydrochloride (Ketamidor^®^, Laboratorios Karizoo S.A.) plus 0.08 mg/kg i.m. medetomidine hydrochloride (Domtor^®^, Ecuphar Veterinaria S.L.U.). The mean anaesthesia induction time was 1.5 min. Anaesthesia was maintained with 1% isoflurane using a paediatric T‐piece system with an APL valve and a mask (Isobavet^®^, Intervet Schering PloughAnimal Health). The O_2_ flow rate was 0.5–1 L/min. After collecting the sperm, anaesthesia was reversed with 0.4 mg/kg of i.m. atipamezole (Antisedan^®^, Ecuphar Veterinaria S.L.U.), an α2‐adrenergic medetomidine antagonist. Animals recovered after 10 min.

Semen was collected twice per week from each animal during the month of June (*n* = 8 collection attempts) using a Lane Pulsator IIIZ electroejaculator (Lane Manufacturing Inc.). Before electrostimulation, the animals were placed in the supine position, and the penis made to protrude for cleaning with a sperm‐washing solution made up of Tris 313.7 mM, citric acid 104.7 mM and glucose 30.3 mM (TCG, 345 mOsm/kg, pH 6.8). It was then maintained protruded in a glass collector (diameter 5.5 mm). A rectal wash was performed by introducing 10 ml of saline solution at 37°C into the rectal cavity with a syringe. The electroejaculation probe (4 mm diameter) was coated with carboxymethyl cellulose gel to improve electrical conductivity, and inserted into the rectum about 4 cm, with the electrodes in the abdominal direction.

Each animal was then subjected to up to four cycles of 14 increasingly strong electrical stimuli, each cycle consisting of two stimuli of 2 V, two of 3 V, two of 4 V, two of 5 V, two of 6 V, two of 7 V and two of 8 V. Each stimulus lasted 4 s with a break of 10 s between stimuli, during which time mild rectal massage was performed with the probe. Stimulation was halted the moment that ejaculation occurred or when four cycles had been completed without ejaculation. Semen was collected in the glass collector. The volume of the ejaculates was measured using a micropipette (Gilson, Villiers Le Bel). Each sample was then diluted 1:1 (v/v) with TEST medium (Irvine Scientific) in a 15 ml Falcon tube at 37°C and quickly transferred in a thermos to an adjacent laboratory. All materials and equipment used to collect, handle and process the semen were either new or sterilized prior to use by autoclaving or by exposure to ultraviolet irradiation, and were maintained at 37°C.

Sperm concentration was determined using a Neubauer chamber (Marienfeld, Lauda‐Königshofen). The vigour of sperm movement was scored subjectively under phase contrast microscopy on a scale from 0 to 5 (0 = no motility; 1 = weak tail movement, no forward progression; 2 = slow forward movement, often in a circular pattern; 3 = moderate forward movement; 4 = rapid forward movement; 5 = very rapid forward movement). Sperm progressive motility was determined using a computer‐aided sperm analysis system (CASA) coupled to a phase contrast microscope (NikonEclipse model 50i; negative contrast) and using a Sperm Class Analyzer v.5.3.0.1 software (Microptic S.L.). Sperm samples were diluted 1:21 (% v/v) in a Tris‐citric acid‐glucose (Sigma Chemical Co.) washing medium (345 mOsm, pH6.8) and loaded onto a warmed (37°C) 20 μm Leja 8‐chamber slide (Leja Products B.V.). Sperm movement characteristics – curvilinear velocity (VCL), straight‐line velocity (VSL), average path velocity (VAP), amplitude of lateral head displacement (ALH) and beat‐cross frequency (BCF)—were then recorded. Three progression ratios, expressed as percentages, were calculated from the velocity measurements described above: linearity (LIN = VSL/VCL × 100), straightness (STR = VSL/VAP × 100) and wobble (WOB = VAP/VCL × 100). Total motility included all sperm cells in motion regardless of the type of movement, whereas progressive motility was considered when STR > 80%. A minimum of three fields and 500 sperm tracks were evaluated at a magnification of 100x for each sample (image acquisition rate 25 frames/s). Sperm viability and acrosomal integrity were analysed by fluorescence microscopy, counting 200 cells, using a combination of propidium iodide (PI; Sigma Chemical Co.) and fluorescein isothiocyanate‐conjugated peanut agglutinin (PNA‐FITC; Sigma Chemical Co.; Soler et al., 2005). Four subpopulations of cells were quantified, i.e. those showing in percentages: (a) intact plasma membrane/intact acrosome, (b) intact plasma membrane/damaged acrosome, (c) damaged plasma membrane/intact acrosome and (d) damage plasma membrane/damage acrosome. The sperm viability percentage was the sum of subpopulations 1 and 2, and the percentage of cells with intact acrosome was the sum of subpopulations 1 and 3.

### 2.3 Semen freezing

Falcon tubes containing collected sperm were place in a beaker with 30 ml water at room temperature and transferred to a refrigerator at 5°C for 1 hr. Once cooled, TEST medium with 12% glycerol (Irvine Scientific) was added to leave a final glycerol concentration of 4%. The sperm suspension was then maintained at 5°C for 2 hr. Sperm was then loaded into 0.25 ml French straws (IMV, L’Aigle, France). The filled straws were sealed by locally generated frictional heat (Ultra seal 21™, Minitube Iberica S.L.) without affecting straw contents by heat transmission. Half of the straws were frozen following a three‐step protocol with a decelerating freezing rate, holding the straws 5 cm above liquid nitrogen bath (a 1.1 L expanded polythene box with a top surface area of 560 cm^2^) for 10 min. The freezing rate pattern was as follows: from 5 to −35°C at 40°C/min, from −35 to −65°C at 17°C/min, and form −65 to −85°C at 3°C/min); they were then plunged into the liquid nitrogen (Esteso et al., 2018); this procedure was standardized to ensure such cooling and checked using a Ventix^®^ K/J/T thermometer (Ventix) equipped with a probe resistant to freezing. The other half underwent a two‐step protocol with an accelerating freezing rate involving the use of a Computer Freezer‐Icetube 1810 freezer unit (Minitüb, Tiefenbach). The freezing rate pattern was as follows: from 5 to −10°C at 5°C/min, and then from −10 to −130°C at 60°C/min.

### 2.4 Semen thawing

After 50–60 days, the frozen semen samples were thawed in a water bath at 37°C for 30 s. The contents of the straws were then decanted into polystyrene tubes and immediately assessed for the semen characteristics described above.

### 2.5 Statistical analysis

Sperm variables that showed non‐normal distributions, as determined using the Shapiro–Wilk test, were arcsine‐transformed (motility, viability, acrosome integrity) or arcsinh‐transformed (CASA variables) before analysis. The effects of the cryopreservation protocols on sperm quality were compared using one‐way ANOVA. All statistical analyses were performed using Statistica software for Windows v.13.0 (StatSoft Inc.).

## 3 RESULTS

The animals responded to the semen collection protocol in a variable manner. In one male, semen was obtained during only three of the eight attempts, while two animals provided semen on all eight occasions. A total of 32 ejaculates were collected, 25 in the first cycle, three in the second cycle and four in the third/fourth cycles (Table 1).

**TABLE 1:** Sperm variables, number of electroejaculation cycles and voltage used with the five animals over the eight collection sessions. Each full cycle consisted of 14 increasingly strong electrical stimuli: two of 2 V, two of 3 V, two of 4 V, two of 5 V, two of 6 V, two of 7 V, and two of 8 V. The cycle number (1–4) and voltage (2–8 V) values refer to the time at which ejaculation occurred

Animal	Collection number	Cycles	Voltage	Volume (µl)	Motility (%)
Ferret 1	1	4		No semen	
	2	4	8 V	55	60
	3	4		No semen	
	4	4		No semen	
	5	4		No semen	
	6	2	4 V	140	10
	7	4		No semen	
	8	1	7 V	30	30
Ferret 2	1	2	3 V	1,000	0
	2	1	6 V	281	5
	3	1	5 V	175	10
	4	1	4 V	50	50
	5	3	8 V	30	70
	6	3	7 V	60	55
	7	2	5 V	No semen	
	8	1	7 V	236	15
Ferret 3	1	1	4 V	135	50
	2	1	4 V	360	55
	3	1	5 V	115	50
	4	1	6 V	325	60
	5	1	6 V	160	50
	6	1	6 V	27	45
	7	1	5 V	60	70
	8	1	5 V	40	60
Ferret 4	1	1	8 V	81	0
	2	3	7 V	70	70
	3	4		No semen	
	4	2	6 V	570	40
	5	1	3 V	40	60
	6	1	3 V	10	80
	7	1	6 V	80	65
	8	4		No semen	
Ferret 5	1	1	8 V	254	5
	2	1	8 V	350	5
	3	1	8 V	100	5
	4	1	6 V	250	5
	5	1	6 V	700	5
	6	1	6 V	390	5
	7	1	5 V	250	5
	8	1	7 V	700	5

The mean ejaculate volume and sperm concentration were 222 ± 41 μl and 88.8 ± 16×10^6^ spz/ml (mean ± *SE*) respectively. The fresh semen had 38 ± 4% motile spermatozoa. The collected semen was characterized by low vigour sperm motility (Table 2). The mean values in the fresh semen for progressive sperm motility (PM) and non‐progressive sperm motility (NPM) were 4.3 ± 1.1% and 27.4 ± 3.7% respectively (Table 2).

**TABLE 2:** Characteristics of the frozen‐thawed domestic ferret sperm (mean ± *SE*)

	Fresh samples	Three‐step decelerating protocol	Two‐step accelerating protocol
Motility (%)	38.5 ± 4.9^a^	0.69 ± 0.42^b^	1.16 ± 0.36^b^
Vigor movement (score 0–5)	1.12 ± 0.13^a^	0.54 ± 0.21^b^	0.38 ± 0.09^b^
Static (%)	68.2 ± 4.1^a^	94.9 ± 1.1^b^	92.9 ± 3.9^b^
Non‐progressive motility (%)	27.4 ± 3.7^a^	4.9 ± 1.0^b^	7.0 ± 1.5^b^
Progressive motility (%)	4.3 ± 1.1^a^	0.2 ± 0.1^b^	0.1 ± 0.05^b^
VCL (μm/s)	44.1 ± 6.6^a^	10.8 ± 2.6^b^	10.0 ± 2.1^b^
VSL (μm/s)	13.2 ± 2.1^a^	2.9 ± 0.8^b^	2.8 ± 0.6^b^
VAP (μm/s)	20.1 ± 3.1^a^	5.4 ± 1.4^b^	4.6 ± 1.0^b^
LIN (%)	25.5 ± 1.9^a^	21.3 ± 4.1^b^	18.8 ± 3.1^b^
STR (%)	55.5 ± 4.1^a^	40.6 ± 5.6^b^	46.1 ± 6.6^b^
WOB (%)	41.1 ± 2.8^a^	38.4 ± 5.2^b^	30.8 ± 4.7^b^
ALH (μm)	2.3 ± 0.3^a^	0.10 ± 0.06^b^	0.25 ± 0.12^b^
BCF (Hz)	4.7 ± 0.7^a^	0.46 ± 0.27^b^	0.71 ± 0.37^b^
Sperm viability (%)	74.5 ± 2.3^a^	29.7 ± 3.7^b^	43.3 ± 3.5^c^
Acrosome integrity (%)	92.9 ± 3.0^a^	58.8 ± 3.4^b^	71.2 ± 3.4^c^

Note

Different lowercase letters between columns indicate significant differences (*p* < .05).

Abbreviations: ALH, amplitude of lateral head displacement; BCF, beat‐cross frequency; LIN, linearity; NAR, normal apical ridge; STR, straightness; VAP, average path velocity; VCL, curvilinear velocity; VSL, straight‐line velocity; WOB, wobble.

After thawing, all sperm variables were poorer than for the fresh ejaculates, irrespective of the freezing method followed (Table 2). The two‐step accelerating protocol returned better values for sperm viability and acrosome status than the three‐step decelerating protocol (*p* < .05; Table 2). No other differences between the freezing methods were seen.

## 4 DISCUSSION

Domestic species can provide models for developing reproductive technologies for phylogenetically related wild species (Wildt et al., 2010). Although specific cryopreservation protocols are commonly required—a reflection of species uniqueness in terms of sperm structure, shape and volume, organelle size, membrane fluidity, the cholesterol/phospholipid ratio and osmotic tolerance (Bóveda et al., 2018; Meryman, 1970), the ejaculated spermatozoa of closely related mustelid species share many motion characteristics (van der Horst et al., 2009). Improved sperm cryopreservation protocols for domestic ferrets might also better preserve the sperm of European mink.

The present results agree with those reported by Esteso et al. (2018) in ibexes, in which the same three‐step decelerating protocol caused greater cryodamage to the sperm cells than a three‐step accelerating protocol. In decelerating methods, the initial high cooling rate achieved with nitrogen vapour—i.e. 40°C/min from 5 to −35°C—involves exposure to the harmful effects of unfrozen, extracellular, hypertonic solutions over the critical −5 to −15°C range when ice growth occurs (Galarza et al., 2019). In addition, such exposure could lead to ‘cold shock’ which, along with any ice crystals formed, could cause injury to sperm cell membranes (Hammadeh et al., 2001; Mazur, 1984). Indeed, the present results show that the two‐step accelerating freezing rate was less harmful to the plasma membrane and acrosome.

The frozen‐thawed sperm viability and acrosome integrity values obtained with the two‐step accelerating protocol were equal to or greater than those published previously for ferrets (van der Horst et al., 2009; Leigh, 2010). With this method, the cooling rate of 60°C/min around the point of ice nucleation might have caused less cryodamage as exposure to the negative effects of unfrozen extracellular hypertonic solutions would have been reduced (Galarza et al., 2019). Moreover, the sperm may have become less shrunken and dehydrated.

Using ram sperm, the same two‐step accelerating protocol used in the present work was compared with a three‐step decelerating and a three‐step accelerating rate protocol, and returned better post‐thaw survival and functionality results than either of the latter two (Galarza et al., 2019). The authors of that study indicate the accelerating two‐step cooling rate to cause less damage to mitochondrial membranes, allowing for better motility than the other protocols (Galarza et al., 2019). However, in the present work, no differences were seen between the tested protocols in terms of motility variables. This might be explained by the poor sperm vigour of the fresh semen (score 1.12 out of a maximum of 5). Certainly, ferret sperm is quite sensitive to osmotic stress (Santymire et al., 2006), to high proportions of seminal plasma, and to urine contamination (Marco‐Jiménez et al., 2005), and the present sample may have suffered from the last two problems: while the mean ejaculate volume was greater than that reported by other authors (222 µl vs. 40–160 µl; Howard et al., 1991; Wildt et al., 1989) the sperm concentration was much lower (only a few ejaculates reached 150‐200 × 10^6^ spermatozoa/ml compared with the 700 × 10^6^ spermatozoa/ml recorded by the above authors). The urine contamination causes an increase in pH, osmolarity (Ellerbrock et al., 2018) and increases the presence of cell debris and other dead cells (Skidmore et al., 2018). All these effects may affect the kinetic activity of the sperm cell. Although dilution with the extender of the seminal sample mitigates the damage caused by urine in fresh semen, its presence might affect progressive movement after freezing‐thawing (Ellerbrock et al., 2018). On the other hand, electrical stimuli also alter the secretion of the accessory sex glands, altering the quantity and composition of the seminal plasma. Variations in seminal plasma have been shown to affect motility before and after freeze‐thaw (Aurich et al., 1996, 2020). Furthermore, the increase in volume due to both causes also exerts a dilution effect that mainly affects motility after freezing. (Nicollas et al., 2011; Peris et al., 2007). To produce a seminal plasma‐free suspension of highly motile sperm devoid of urine and cell debris should be a priority, and thus the inclusion of selective washing techniques (Santiago‐Moreno et al., 2014) in the semen management protocol of these species could be recommended. Future work should check whether the method of sperm collection used is associated with urine contamination or excess seminal plasma.

A TEST diluent (Irvine Scientific) was used in the present study because it seems to provide an adequate protection according a previous report (van der Horst et al., 2009). However, a certain influence of the characteristics of diluent and the type and the percentage of cryoprotectant (4% glycerol in our study) on the motility data after freezing should not be ruled out. Alternative diluents (e.g. milk based extenders), glycerol concentrations and even other permeant cryoprotectant should be explored in this species. In this regard, while some species do not tolerate exposure to 4%–8% of glycerol concentrations (e.g. porcine sperm), in other species in which cryopreservation is problematic (e.g. the marsupials kangaroos and wallabies) there is a considerable benefit from increasing the glycerol concentrations to levels (15%) which would be normally be considered high (Holt, 2000).

Inter‐individual differences may affect sperm freezability (Holt, 2000). The greater or lesser response may be conditioned by the age. In previous studies (Wildt et al., 1989 and Howard et al., 1991) ferrets of 24–36 months were used; thus we considered that the 27 months was an adequate sampling period. Although the ferrets reach the puberty at 12 months, we cannot rule out the role of age on sperm response to the freezing‐thawing protocols. In addition, the conditioning to the facilities might be an important factor, and maybe the ferrets were not adequately acclimatized after 5 weeks; stress might modify endocrine status (e.g. testosterone levels) that influences sperm cryoresistance (Martínez‐Fresneda et al., 2019). Santymire et al. (2019) found that inbreeding mainly affects the percentages of motile sperm, progressive motile sperm and in the presence of abnormal forms. We do not have genetic data, but consanguinity cannot be entirely excluded because the animals come from the same commercial breeder.

Howard et al. (1991) showed a close relationship to exist between post‐thaw motility and acrosome integrity in ferret sperm, and Pukazhenthi et al. (2007) suggest acrosome status to be a potentially useful indicator of sperm function and fertilization potential. Despite the poor motility of the present frozen‐thawed sperm (obtained with either protocol) the adequate acrosome integrity and sperm viability values recorded with the two‐step accelerating protocol suggest that samples thus cryopreserved might provide acceptable fertility results after intrauterine or intraoviductal artificial insemination.

Most studies on ferret semen have involved collection based on the electroejaculation protocol developed by Wildt et al. (1989). However, Aulerich et al. (1972) developed a method for American mink (*Neovison vison*) semen collection that required fewer impulses, thus providing benefits in terms of animal welfare. Recently, transrectal ultrasound‐guided massage of the accessory sex glands (TUMASG method) has been reported a useful alternative to conventional electroejaculation, reducing and even obviating the need for electrical stimuli in wild and domestic ruminants (Santiago‐Moreno et al., 2013; Ungerfeld et al., 2015, 2016). In the present work, 78% of ejaculations were achieved with no more than 14 electrical impulses. Discounting the animals that failed to reach ejaculation, the maximum number of impulses required was 32 compared with the 100 required by other authors in domestic ferrets and other mustelids (van der Horst et al., 2009; Santymire et al., 2007; Wildt et al., 1989). Thus, electrical stimuli combined with transrectal massage would appear to allow an overall reduction in the number of electrical stimuli required, but experimental comparisons are required to confirm this.

In conclusion, combining rectal massage with electroejaculation reduces the number of electrical stimuli required for ejaculation to occur. Although the protocol with accelerating freezing rate was associated with better post‐thaw sperm viability and acrosome integrity, neither of the two freezing methods tested provided adequate motility results after thawing.

## CONFLICT OF INTEREST

None of the authors has any financial and personal relationships with other people or organizations that could inappropriately influence (bias) their work. The authors have no conflict of interest to declare.

## ETHICAL APPROVAL DETAILS

All animals were managed in a manner consistent with the Spanish Policy for Animal Protection (RD 53/2013), which conforms to European Union Directive 2010/63/EU regarding the protection of animals used in scientific experiments.

## AUTHOR CONTRIBUTION

Adolfo Toledano: Data curation; Investigation; Writing‐original draft. Cristina Castaño: Data curation; Investigation; Resources. Rosario Velazquez: Data curation; Software. Paula Boveda: Investigation; Resources. Antonio Lopez‐Sebastian: Conceptualization; Writing‐review & editing. Eva Martinez Nevado: Writing‐review & editing. Silvia Villaverde‐Morcillo: Methodology; Validation. Milagros C Esteso: Conceptualization; Funding acquisition. J. Santiago‐Moreno: Conceptualization; Formal analysis; Investigation; Writing‐review & editing.

### PEER REVIEW

The peer review history for this article is available at https://publons.com/publon/10.1002/vms3.362.

## Supporting information

Supplementary Material

## Data Availability

The data that support the findings of this study are available in the Supplementary Material S1 of this article.
