# Supplementary material for: Cryopreservation of ferret (Mustela putorius furo) sperm collected by rectal massage and electroejaculation: Comparison of a decelerating and an accelerating freezing rate protocol
Source: Vet Med Sci. 2020 Oct 11;7(1):256–63. doi: 10.1002/vms3.362 (PMC7840209; doi:10.1002/vms3.362)
Supplement: Supplementary file 1 — Supplementary Material [file VMS3-7-256-s001.pdf]

Additional Table 1: Monitoring data during the electroyaculation, scrotal circunference and body weight of the five domestic ferrets over the eight collection sessions..

Date (dd/mm/yyyy)	ID	Anesthetic induction time (min)	Heart rate beats per minute	% Oxygen saturation	Scrotal circumference left testicle (cm)	Scrotal circumference right testicle (cm)	Body weight (g)
04/06/2019	H1	2	180-190	100	1,2	1,2	
04/06/2019	H2	1,5	200-215	100	1	1	
04/06/2019	H3	2	180-190	100	1,2	1,1	
04/06/2019	H4	2	105-209	95	1,2	1,3	907
04/06/2019	H5	2	218-238	90-98	1,2	1,2	1120
06/06/2019	H2	1,45					
06/06/2019	H3	1,5	186-173	100-98			
06/06/2019	H4	2	180-200	100			
06/06/2019	H5	2,15	212	100			
06/06/2019	H1	1,45	166-224	99			
11/06/2019	H3	1	191	97	1,1	1,2	959
11/06/2019	H4	1,15	168	100	1,3	1,2	863
11/06/2019	H5			99	1,1	1,1	1300
11/06/2019	H1		164	100	1,1	1,15	1119
11/06/2019	H2			100	1,2	1,2	1090
13/06/2019	H4	2	100-184	100			
13/06/2019	H5	1	206	100			
13/06/2019	H1	1,15					
13/06/2019	H2		182	100			
13/06/2019	H3	1,5	188	100			
18/06/2019	H5	2,2	46-225	100	1,2	1,2	1282
18/06/2019	H1	1,3	275	100	1,2	1,1	1131
18/06/2019	H2	1,17		100	1,25	1,2	1058
18/06/2019	H3	1,5					908
18/06/2019	H4				1,2	1,2	841
20/06/2019	H1	1,5					
20/06/2019	H2	1,15					
20/06/2019	H3				1,2	1,2	
20/06/2019	H4	1,5					
20/06/2019	H5	2,1					
25/06/2019	H2	2,15			1,2	1,1	
25/06/2019	H3	1,5			1,2	1,2	
25/06/2019	H4	2			1,15	1,15	
25/06/2019	H5	2			1,3	1,2	
25/06/2019	H1				1,2	1,2	
27/06/2019	H3	2					
27/06/2019	H4	1,15					
27/06/2019	H5	1,15					
27/06/2019	H1	1,15					
27/06/2019	H2	1,15					

Additional Table 2: Characteristics of the fresh sperm of the samples obtaained from the five domestic ferrets in the eight collection sessions.

Date (dd/mm/yyyy)	ID	Sperm Vol. (µl)	Motility (%)	Score (0-5)	Concentration (mill/ml)	Sperm viability (%)	Acrosomal integrity (%)
04/06/2019	H1	NO SEMEN					
04/06/2019	H2	1000	0	0	8	28	97
04/06/2019	H3	135	50	2,5	160	11	98
04/06/2019	H4	81	0	0	300	13	100
04/06/2019	H5	254	<5	0,5	16	41	92
06/06/2019	H2	281	5	0,5	4	26	99
06/06/2019	H3	360	55	2	150	28	96
06/06/2019	H4	70	70	2	160	2	100
06/06/2019	H5	350	5	0,5	8	35	97
06/06/2019	H1	55	60	2	80	28	96
11/06/2019	H3	115	50	1,5	120	21	99
11/06/2019	H4	NO SEMEN					
11/06/2019	H5	100	5	1	60	16	99
11/06/2019	H1	NO SEMEN					
11/06/2019	H2	175	10	0,5	40	45	89
13/06/2019	H4	570	40	1	40	31	95
13/06/2019	H5	250	5	0,5	100	58	95
13/06/2019	H1	NO SEMEN					
13/06/2019	H2	50	50	1	140	30	96
13/06/2019	H3	325	60	2	16	21	96
18/06/2019	H5	700	<5	0,5	4	44	94
18/06/2019	H1	NO SEMEN					
18/06/2019	H2	30	70	2	140	12	99
18/06/2019	H3	160	50	1,5	8	12	99
18/06/2019	H4	40	60	2,5	140	19	92
20/06/2019	H1	140	10	0,5	20	44	94
20/06/2019	H2	60	55	1	80	12	97
20/06/2019	H3	27	45	1	28	10	100
20/06/2019	H4	10	80	3		36	98
20/06/2019	H5	390	5	0,5	4	0	0
25/06/2019	H2	NO SEMEN					
25/06/2019	H3	60	70	1,5	160	10	99
25/06/2019	H4	80	65	1	200	14	80
25/06/2019	H5	250	<5	0,5	4,8	31	95
25/06/2019	H1	NO SEMEN					
27/06/2019	H3	40	60	1,5	420	18	96
27/06/2019	H4	NO SEMEN					
27/06/2019	H5	700	<5	0,5	4	27	100
27/06/2019	H1	30	30	0,5	100	25	94
27/06/2019	H2	236	15	0,5	40	22	93

Additional Table 3: Sperm progressive motility by computer-aided sperm analysis system (CASA) of the fresh domestic ferret sperm .

IDENTIFICATION	Static (%)	Non-progressive motility (%)	Progressive motility (%)	VCL	VSL	VAP	LIN	STR	WOB	ALH	BCF
H1 20-6-19 fresco 1.60	96,6	3,4	0,0	0,0	0,0	0,0	0,0	0,0	0,0	0,0	0,0
H1 27-6-19 fresco 1.1	32,5	64,0	3,5	39,4	11,0	20,6	28,0	53,7	52,2	3,8	9,7
H1 6-6-19 fresco	72,9	23,1	4,0	48,6	18,3	24,0	37,7	76,3	49,5	4,2	7,9
H2 11-6-19 fresco 1.1	70,2	29,8	0,0	18,0	5,3	8,9	29,6	59,7	49,6	0,0	0,0
H2 13-6-19 fresco 1.1	58,6	40,6	0,8	24,2	8,2	13,4	33,8	61,0	55,5	2,0	7,4
H2 18-6-19 fresco 1.33	69,6	14,3	16,1	119,8	37,0	48,2	30,9	76,8	40,3	6,7	9,0
H2 20-6-19 fresco 1.60	78,0	15,0	7,0	83,1	21,5	31,1	25,9	69,1	37,5	4,0	7,3
H2 27-6-19 fresco 1.50	95,8	2,1	2,1	0,0	0,0	0,0	0,0	0,0	0,0	0,0	0,0
H2 6-6-19 fresco	63,8	36,2	0,0	17,7	4,0	8,0	22,8	50,3	45,3	0,0	0,0
H3 11-6-19 fresco 1.1	64,5	33,9	1,6	24,5	7,9	12,2	32,3	64,9	49,8	2,2	7,7
H3 13-6-19 fresco 1.1	32,1	60,8	7,0	53,9	16,3	27,4	30,2	59,3	50,9	3,4	7,4
H3 18-6-19 fresco 1.1	41,5	56,6	1,8	31,8	9,0	15,6	28,1	57,3	49,1	3,6	8,6
H3 20-6-19 fresco 1.60	71,6	17,9	10,4	76,7	22,2	30,2	28,9	73,4	39,4	2,9	4,0
H3 25-6-19 fresco 1.17	75,9	19,1	5,0	66,7	16,6	24,3	24,9	68,2	36,5	5,2	8,8
H3 27-6-19 fresco 1.50	23,4	70,3	6,3	86,1	19,3	41,5	22,5	46,5	48,3	3,6	6,1
H3 4-6-19 fresco	77,0	20,6	2,4	56,5	17,5	26,1	31,0	67,1	46,1	4,4	9,8
H3 6-6-19 fresco 1.1	48,8	49,4	1,8	29,4	11,3	16,1	38,4	70,2	54,7	1,7	6,6
H4 13-6-19 fresco 1.1	87,3	12,7	0,0	18,3	3,6	7,5	19,7	47,9	41,1	0,0	0,0
H4 18-6-19 fresco 1.33	64,9	20,9	14,2	98,7	31,5	41,9	31,9	75,2	42,5	5,4	6,5
H4 20-6-19 fresco 1.60	16,2	58,1	25,7	129,6	44,5	74,0	34,4	60,2	57,1	5,4	10,3
H4 25-6-19 fresco 1.17	59,3	31,9	8,8	54,4	19,8	26,8	36,3	74,0	49,1	1,5	3,5
H4 6-6-19 fresco 1.17	89,5	6,8	3,7	84,5	30,4	36,9	35,9	82,2	43,7	4,1	6,6
H5 11-6-19 fresco 1.20	88,6	9,1	2,3	41,2	11,2	16,9	27,1	66,1	41,0	2,2	7,7
H5 13-6-19 fresco 1.1.	93,6	6,4	0,0	15,4	1,4	5,2	9,3	27,7	33,5	0,0	0,0
H5 18-6-19 fresco 1.1	86,5	13,5	0,0	13,7	3,4	6,2	24,4	54,3	45,0	0,0	0,0
H5 20-6-19 fresco 1.60	78,4	21,6	0,0	9,9	2,6	4,5	26,2	57,6	45,5	0,0	0,0
H5 25-6-19 fresco 1.17	98,6	1,4	0,0	0,0	0,0	0,0	0,0	0,0	0,0	0,0	0,0
H5 27-6-19 fresco 1.1	65,0	35,0	0,0	16,0	3,2	6,5	19,8	49,0	40,4	0,0	0,0
H5 6-6-19 fresco 1.1	79,0	20,6	0,4	22,0	6,6	10,6	30,0	62,4	48,1	0,3	2,5

Additional table 4: . Sperm characteristics of each frozen-thawed seminal domestic ferret sample

ID	DATE	Freezing rate	Motility (%)	Score (0-5)	Sperm viability (%)	Acrosomal integrity (%)
H1	20/06/2019	Accelerating	0	0	75	77
H2	04/06/2019	Accelerating	0	0	87	18
H2	06/06/2019	Accelerating	0	0	66	67
H2	11/06/2019	Accelerating	0	0	61	72
H2	13/06/2019	Accelerating	0	0	67	55
H2	20/06/2019	Accelerating	0	0	89	69
H2	27/06/2019	Accelerating	5	0,5	85	40
H3	04/06/2019	Accelerating	0	0	60	61
H3	06/06/2019	Accelerating	0,5	0,5	80	77
H3	11/06/2019	Accelerating	2	1	55	82
H3	13/06/2019	Accelerating	1	1	79	84
H3	18/06/2019	Accelerating	1	1	75	88
H3	20/06/2019	Accelerating	5	1	84	89
H3	25/06/2019	Accelerating	5	1	67	88
H4	06/06/2019	Accelerating	1	0,5	69	89
H4	13/06/2019	Accelerating			0	0
H4	18/06/2019	Accelerating	5	1	73	87
H4	25/06/2019	Accelerating	1	1	73	78
H5	04/05/2019	Accelerating	0	0	42	72
H5	06/06/2019	Accelerating	0	0	60	72
H5	11/06/2019	Accelerating	2	0,5	77	88
H5	13/06/2019	Accelerating	0	0	67	52
H5	18/06/2019	Accelerating	0	0	82	66
H5	20/06/2019	Accelerating	0	0	84	75
H5	25/06/2019	Accelerating	0	0	56	81
H5	27/06/2019	Accelerating	0,5	0,5	84	52
H1	06/06/2019	Decelerating	5	1	82	68
H1	20/06/2019	Decelerating	0	0	84	68
H1	27/06/2019	Decelerating	0,5	0,5	86	71
H2	04/06/2019	Decelerating	0	0	78	35
H2	06/06/2019	Decelerating	0	0	59	57
H2	11/06/2019	Decelerating	0	0	60	44
H2	13/06/2019	Decelerating	0,5	0,5	80	70
H2	18/06/2019	Decelerating	2	1	84	53
H2	27/06/2019	Decelerating	1	5	88	63
H3	04/06/2019	Decelerating	0	0	74	43
H3	06/06/2019	Decelerating	1	0,5	76	93
H3	11/06/2019	Decelerating	1	0,5	53	64
H3	13/06/2019	Decelerating	1	1	82	73
H3	18/06/2019	Decelerating	1	0,5	45	88
H3	27/06/2019	Decelerating	10	2	60	75
H4	04/06/2019	Decelerating	0	0	76	35
H4	17/06/2019	Decelerating			65	68
H4	20/06/2019	Decelerating	0	0	60	55
H5	04/06/2019	Decelerating	0	0	64	74
H5	06/06/2019	Decelerating	0	0	62	52
H5	11/06/2019	Decelerating	0,5	0,5	90	22
H5	13/06/2019	Decelerating	0	0	65	50
H5	18/06/2019	Decelerating	0	0	68	52
H5	20/06/2019	Decelerating	0	0	61	38
H5	25/06/2019	Decelerating	0	0	75	40
H5	27/06/2019	Decelerating	0,5	0,5	58	80

Additional Table 5: Sperm progressive motility by computer-aided sperm analysis system (CASA) of each frozen-thawed seminal domestic ferret sample

ID	Freezing rate	Static (%)	Non-progressive motility (%)	Progressive motility (%)	VCL	VSL	VAP	LIN	STR	WOB	ALH	BCF
H1 BIO 20-6-19	Accelerating	99,4	0,6	0,0	0,0	0,0	0,0	0,0	0,0	0,0	0,0	0,0
H2 BIO 11-6-19	Accelerating	97,8	2,2	0,0	8,7	3,5	4,8	40,5	74,1	54,7	0,0	0,0
H2 BIO 13-6-19	Accelerating	98,6	1,4	0,0	7,2	0,7	0,8	9,7	85,0	11,4	0,0	0,0
H2 BIO 20-6-19	Accelerating	100,0	0,0	0,0	0,0	0,0	0,0	0,0	0,0	0,0	0,0	0,0
H2 BIO 4-6-19	Accelerating	100,0	0,0	0,0	0,0	0,0	0,0	0,0	0,0	0,0	0,0	0,0
H2 BIO6-6-19	Accelerating	98,2	1,8	0,0	2,0	0,3	0,4	13,4	74,5	18,0	0,0	0,0
H2 BIO 27_06_19	Accelerating	95,7	4,3	0,0	3,3	0,6	1,4	17,0	40,7	41,8	0,0	0,0
H3 BIO 11-6-19	Accelerating	92,9	7,1	0,0	19,8	5,0	7,9	25,3	63,1	40,0	0,0	0,0
H3 BIO13-6-19	Accelerating	78,4	20,7	0,9	20,2	7,1	9,8	35,1	72,3	48,6	0,5	2,3
H3 BIO 18-5-19	Accelerating	86,2	13,8	0,0	29,3	5,3	11,2	18,0	47,0	38,4	0,0	0,0
H3 BIO 20-6-19	Accelerating	82,8	16,8	0,4	23,7	7,4	11,9	31,2	62,1	50,2	0,5	1,2
H3 BIO 25-6-19	Accelerating	84,3	14,8	0,9	25,6	8,4	12,9	32,6	65,0	50,2	2,5	8,5
H3 BIO 4-6-19	Accelerating	100,0	0,0	0,0	0,0	0,0	0,0	0,0	0,0	0,0	0,0	0,0
H3 BIO 6-6-19	Accelerating	90,1	9,9	0,0	10,2	5,1	6,6	49,6	76,9	64,5	0,0	0,0
H4 BIO 18-6-19	Accelerating	78,3	21,4	0,3	26,9	6,7	12,6	24,9	53,1	46,9	1,7	2,4
H4 BIO 25-6-19	Accelerating	82,8	16,7	0,6	30,2	10,0	16,5	33,3	60,8	54,7	1,0	3,3
H4 BIO 6-6-19	Accelerating	81,8	18,2	0,0	13,1	4,8	6,1	36,8	79,1	46,5	0,0	0,0
H5 BIO 11-6-19	Accelerating	91,6	8,4	0,0	16,5	3,8	7,2	23,2	53,5	43,4	0,0	0,0
H5 BIO13-6-19	Accelerating	99,5	0,5	0,0	0,9	0,1	0,1	7,7	100,0	7,7	0,0	0,0
H5 BIO18-6-19	Accelerating	100,0	0,0	0,0	0,0	0,0	0,0	0,0	0,0	0,0	0,0	0,0
H5 BIO 25-6-19	Accelerating	100,0	0,0	0,0	0,0	0,0	0,0	0,0	0,0	0,0	0,0	0,0
H5 BIO27-6-19	Accelerating	93,3	6,7	0,0	5,6	2,1	3,6	37,6	59,0	63,7	0,0	0,0
H5 BIO 4-6-19	Accelerating	98,7	1,3	0,0	2,7	0,2	0,6	7,7	33,3	23,1	0,0	0,0
H5 BIO 6-6-19 2	Accelerating	91,6	8,4	0,0	3,4	0,0	0,6	0,0	0,0	16,0	0,0	0,0
H5 BIO20_06_19	Accelerating	99,5	0,5	0,0	2,0	0,6	1,0	27,5	54,1	50,8	0,0	0,0
H1 VAP 20-6-19	Decelerating	99,0	1,0	0,0	2,2	0,4	0,5	16,9	74,5	22,7	0,0	0,0
H1 VAP 27-6-19	Decelerating	94,0	6,0	0,0	4,0	1,2	2,0	29,2	57,7	50,6	0,0	0,0
H1 VAP 6-6-19	Decelerating	89,0	10,7	0,3	31,5	12,4	18,9	39,3	65,5	59,9	0,5	2,6
H2 VAP 11-6-19	Decelerating	99,4	0,6	0,0	2,0	0,3	1,0	16,3	33,3	49,0	0,0	0,0
H2 VAP 13-6-19	Decelerating	98,9	1,1	0,0	4,2	0,5	1,0	11,0	44,7	24,5	0,0	0,0
H2 VAP 18-6-19	Decelerating	91,6	8,4	0,0	28,8	7,3	15,7	25,3	46,5	54,5	0,0	0,0
H2 VAP27-6-19	Decelerating	94,1	5,9	0,0	16,4	1,9	8,5	11,8	22,7	52,0	0,0	0,0
H2 VAP 4-6-19	Decelerating	100,0	0,0	0,0	0,0	0,0	0,0	0,0	0,0	0,0	0,0	0,0
H2 VAP 6-6-19	Decelerating	98,9	1,1	0,0	0,0	0,0	0,0	0,0	0,0	0,0	0,0	0,0
H3 VAP 11-6-19	Decelerating	94,2	5,8	0,0	16,5	5,9	8,3	35,8	71,5	50,1	0,0	0,0
H3 VAP 13-6-19	Decelerating	79,9	20,1	0,0	36,1	6,6	15,8	18,4	42,1	43,6	0,0	0,0
H3 VAP 18-6-19	Decelerating	87,6	12,4	0,0	15,7	1,7	4,9	10,6	33,9	31,3	0,0	0,0
H3 VAP 27-6-19	Decelerating	79,0	17,1	3,8	37,5	15,2	22,5	40,5	67,4	60,0	1,3	5,4
H3 VAP 4-6-19	Decelerating	99,7	0,3	0,0	7,6	0,8	3,9	10,8	21,2	50,9	0,0	0,0
H3 VAP 6-6-19.	Decelerating	92,6	6,7	0,7	43,3	12,1	20,1	27,8	59,9	46,5	0,8	4,0
H4 VAP 17-6-19	Decelerating	98,5	1,5	0,0	0,0	0,0	0,0	0,0	0,0	0,0	0,0	0,0
H4 VAP 20-6-19	Decelerating	96,4	3,6	0,0	1,7	0,6	1,0	33,2	58,9	56,4	0,0	0,0
H4 VAP4-6-19	Decelerating	99,7	0,3	0,0	0,0	0,0	0,0	0,0	0,0	0,0	0,0	0,0
H5 VAP11-6-19	Decelerating	94,7	5,3	0,0	15,7	4,6	7,0	29,0	64,6	44,8	0,0	0,0
H5 VAP 13-6-19	Decelerating	98,5	1,5	0,0	8,2	0,7	2,6	9,1	29,0	31,3	0,0	0,0
H5 VAP 18-6-19	Decelerating	96,3	3,7	0,0	1,1	0,8	0,9	68,8	86,9	79,2	0,0	0,0
H5 VAP 20-6-19	Decelerating	99,6	0,4	0,0	0,0	0,0	0,0	0,0	0,0	0,0	0,0	0,0
H5 VAP25-6-19	Decelerating	99,0	1,0	0,0	0,0	0,0	0,0	0,0	0,0	0,0	0,0	0,0
H5 VAP 27-6-19	Decelerating	93,5	6,5	0,0	5,4	1,1	1,8	19,6	57,7	33,9	0,0	0,0
H5 VAP 4-6-19	Decelerating	98,6	1,4	0,0	1,4	1,2	1,4	83,6	87,1	96,0	0,0	0,0
H5 VAP 6-6-19	Decelerating	96,2	3,8	0,0	3,2	0,6	2,0	18,7	29,9	62,6	0,0	0,0
